# Biochar Coating Is a Sustainable and Economical Approach to Promote Seed Coating Technology, Seed Germination, Plant Performance, and Soil Health

**DOI:** 10.3390/plants11212864

**Published:** 2022-10-27

**Authors:** Kangkang Zhang, Zaid Khan, Qing Yu, Zhaojie Qu, Jiahuan Liu, Tao Luo, Kunmiao Zhu, Junguo Bi, Liyong Hu, Lijun Luo

**Affiliations:** 1 MARA Key Laboratory of Crop Ecophysiology and Farming System in the Middle Reaches of the Yangtze River, College of Plant Science and Technology, Huazhong Agricultural University, Wuhan 430070, China; 2 Shanghai Agrobiological Gene Center, No. 2901 Beidi Road, Shanghai 201106, China; 3 College of Natural Resources and Environment, South China Agricultural University, Guangzhou 510642, China; 4 Hubei Key Laboratory of Nutritional Quality and Safety of Agro-products, Institute of Quality Standard and Testing Technology for Agro-Products, Hubei Academy of Agricultural Sciences, Wuhan 430072, China

**Keywords:** biochar, seed coating, seed enhancement technology, review, seed vigor, seed germination

## Abstract

Seed germination and stand establishment are the first steps of crop growth and development. However, low seed vigor, improper seedbed preparation, unfavorable climate, and the occurrence of pests and diseases reduces the germination rate and seedling quality, resulting in insufficient crop populations and undesirable plant growth. Seed coating is an effective method that is being developed and applied in modern agriculture. It has many functions, such as improving seed vigor, promoting seedling growth, and reducing the occurrence of pests and diseases. Yet, during seed coating procedures, several factors, such as difficulty in biodegradation of coating materials and hindrance in the application of chemical ingredients to seeds, force us to explore reliable and efficient coating formulations. Biochar, as a novel material, may be expected to enhance seed germination and seedling establishment, simultaneously ensuring agricultural sustainability, environment, and food safety. Recently, biochar-based seed coating has gained much interest due to biochar possessing high porosity and water holding capacity, as well as wealthy nutrients, and has been proven to be a beneficial agent in seed coating formulations. This review presents an extensive overview on the history, methods, and coating agents of seed coating. Additionally, biochar, as a promising seed coating agent, is also synthesized on its physico-chemical properties. Combining seed coating with biochar, we discussed in detail the agricultural applications of biochar-based seed coating, such as the promotion of seed germination and stand establishment, the improvement of plant growth and nutrition, suitable carriers for microbial inoculants, and increase in herbicide selectivity. Therefore, this paper could be a good source of information on the current advance and future perspectives of biochar-based seed coating for modern agriculture.

## 1. Introduction

Farmers have long pursued crop seeds with high vigor to guarantee increased and uniform germination post-sowing, along with successful plant establishment, which contributes to achieving higher yield [1]. Yet, the undesirable properties of initial seeds, i.e., hardness, low seed vigor, and seed aging [2], result in erratic germination and poor stand establishment. Additionally, there are many external factors for seedling suboptimal germination and mortality, including improper farming operations (unsuitable sowing depth, uneven soil, the excessive use of fertilizers in the seedbed) and a wide range of environmental stresses (drought, chill) and biological stresses (weed infestation, animal and insect damage) [3]. It is difficult to change and regulate environmental conditions in a large area. Therefore, the judicious application of seed treatments may provide a promising attempt to improve seed vigor, seedling stand, and stress resistance.

In the past few years, seed enhancement technologies, including seed priming and seed coating, have been employed to boost seed performance by suffering from specific conditions and regimes [4,5]. Seed enhancements are most effective when they are goal-oriented and plant-specific to assure optimal seedling establishment and promote production under changing climatic environments [4]. Recent studies reported that seed enhancement techniques produced a healthy crop stand and, additionally, improved the growth and physio-morphological traits of the plant [6]. The application of seed priming is relatively common among different seed treatment technologies. Seed priming, known as the earliest seed treatment technique, refers to on-farm seed priming whereby seeds are controlled and hydrated in either water or water solution with additives or through contact with a solid carrier in order to start the germination while preventing radicle emergence [7]. Seed priming resulted in synchronized emergence, and uniform stand establishment due to a decline in the lag time to imbibition and a better repair of metabolic segments and osmotic adjustment [8,9]. Nevertheless, seed priming is facing many challenges, such as product conservation, viability losses during storage, and difficulties in upscaling management [2,10]. Moreover, this makes the process time-consuming and cumbersome when priming large quantities of seeds that require subsequent drying.

Seed enhancements via seed coating may overcome these issues. Seed coating technology has been considered a promising approach to alleviate the pressure of agricultural activities and combat abiotic and biotic stresses. Seed coating is a reliable technique to apply external materials in close proximity to germinating seeds, which ultimately increases seed quality and yield via improving seed placement and performance [11]. Some of these exogenous materials include the application of nutrients, plant growth regulators, and microorganisms [12], which positively influence seed germination, emergence, and early seedling growth [4]. Likewise, the utilization of non-active or active materials into seeds during seed coating alters the original shape and size of seeds, thereby enhancing a build-up in seed weight, which facilitates seed handling and sowing. Recently, a biomaterial based on silk and trehalose has been mixed with rhizobacterial through a seed coating approach, which boosted seed germination and mitigated soil salinity [3]. The seed coating technique has been used as an efficient tool for raising plant establishment and the development of wheat under water deficit conditions [13].

In recent years, biochar research has received increasing attention due to the unique structure and characteristics of biochar. Biochar derived from biomass through the pyrolysis process is usually employed as a soil amendment [14]. Previously, considerable studies reported that biochar application helps to improve soil properties, increase crop productivity, and enhance carbon sequestration [15,16]. However, the handling of biochar faces one of the major barriers of the use process due to its low energy density and bulk density, along with expensive transporting and storing costs Additionally, the wide utilization of biochar results in the generation of dust, which could lead to product loss and harm the health of workers. It is potentially possible to resolve the above-mentioned difficulties by biochar modification, such as biochar-based seed coating technology. Coating techniques have strong potential toward increasing the value of biochar, as well as its economic viability owing to the high mass and energy density of coatings and their convenient storage. Additionally, it is able to minimize dust generation via the enlargement of particle sizes, which conduces to the environment and human health. Here, the currently published studies on seed coating techniques and the properties and roles of biochar are extensively reviewed. Moreover, by combining seed coating with biochar, we particularly focus on the agricultural applications of biochar-based seed coating. New research opportunities and future prospects are also highlighted.

## 2. The Development and Classification of Seed Coating Technology

### 2.1. The Development of Seed Coating Technology

**Figure 1:**
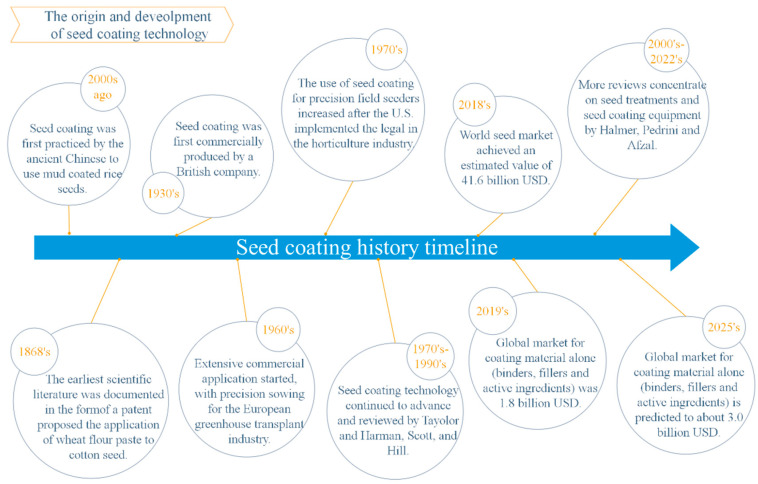
Timeline of the history of seed coating technology.

Seed coating technology is an ancient practice with previous evidence of its utilization being traced back to as far as 2000 years ago by the ancient Chinese, who used mud-coated rice seeds to maintain their dominance in flooded fields (Figure 1) [17]. The first seed patent was filed in 1868 to improve cotton seed for planting by coating a mixture of a glutinous material and gypsum [18], which was the earliest scientific literature on seed coating. Seed coating was first commercially produced for cereal seeds in the 1930s by a British seed company. During the mid-20th century, Jeffs [19] reviewed numerous seed coating techniques that were developed for enhancing agricultural production. The extensive commercial application of seed coating started in the 1960s to increase precision sowing for the greenhouse transplant industry in Europe [20], followed shortly by the United States’ agricultural sector in the 1970s after implementing stricter legal requirements in the horticulture industry [20,21]. Seed coating technologies continually increased from the 1970s to the 1990s, as reviewed by Scott [22], Taylor and Harman [23], and Hill [21].

Since the 21st century, more overviews concentrate on seed enhancements and seed coating equipment by Halmer [24], Pedrini [1,25], and Afzal [26]. In 2018, the world seed market achieved an estimated value of 41.6 billion USD due to the increase in scientific and agricultural prowess [27]. Currently, this technology has proven to be so effective that almost exclusively field crops and horticultural crop seeds are performed, whose market is estimated to reach 18 billion USD each year [28]. With the rapid spread and development of seed coating techniques in developing countries, the global market for materials alone (binders, fillers, and other additives) that are utilized in seed coating was 1.8 billion USD in 2019, which is predicted to rise to almost 3.0 billion USD by 2025 [26,29].

### 2.2. Definition of Modern Seed Coating

Previously, many scientists have proposed a definition of seed coating. Porter [30] suggested that seed coating is used for the application of biostimulants, nutrients, and other products that alleviate biotic and abiotic stresses encountered after sowing. Scott [22] defined seed coating as the application of needed materials to the seed surface that affect the seed or soil at the seed–soil interface. Kaufman [20] proposed that seed coating is the process of applying exogenous materials to the surface of the natural seed coat, which is used for the physical modification of seed or the delivery of active ingredients. However, modern seed coating has a new and broad interpretation. Modern seed coating technology refers to a reliable and efficient technique to apply external materials to cover the natural seed coat, resulting in forming a thinner or thicker layer on the surface. The aims of modern seed coating include achievement in uniform seed dimensions, improvement in seed quality, such as seed vigor and viability, the stimulation of seed germination and seedling growth, and promotion to crop yield via improving seed placement and performance, as well as increase to stress tolerance.

### 2.3. Classification of Seed Coating Methods

Seed coating methods are generally categorized as dependent on the shape, size, weight, and sorting properties of the coated seeds [1,25,31]. Even though the terminology adopted in the literature regarding the type of seed coating is not consistent, the methods most used and reported are seed dressing, film coating, encrusting, pelleting, and extruded pelleting [32,33].

#### 2.3.1. Seed Dressing

**Figure 2:**
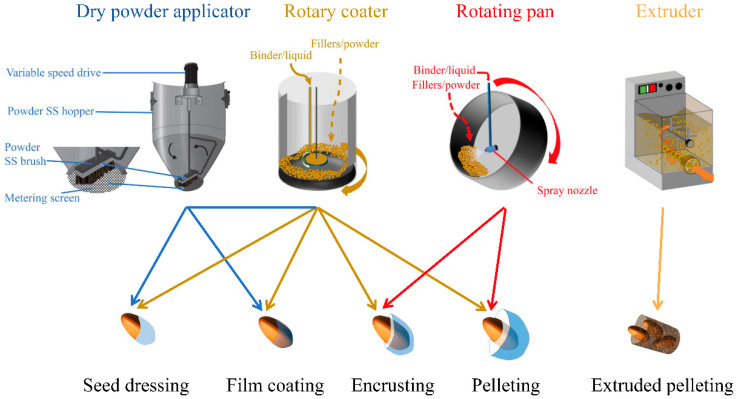
The four main coating equipment and five types of related seed coating. SS: stainless steel.

**Figure 3:**
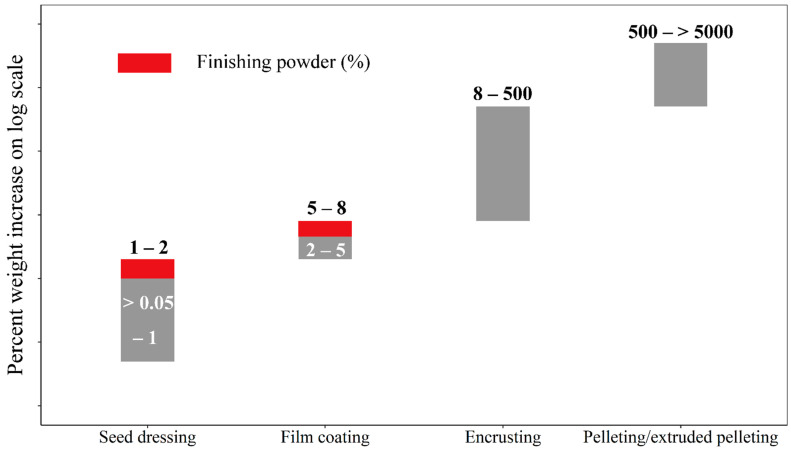
Percent weight increases after five major methods of seed coating. The red for seed dressing and film coating is the application of the finishing powder during the coating process. The percent weight increase is shown on a log scale to aid comparison between methods.

Seed dressing is the most commonly adopted method for active coating ingredients with low dosages onto the surface of seeds [34]. The primary seed coating equipment for seed dressing is a rotary coater (Figure 2), which comprises a metal cylinder with a concave disc, along with two spinning base discs. The concave disk at the bottom drives the seed mass to move steadily along the walls of the cylinder when rotating, while the smaller rotating one attached to the drum lid and suspended in the middle of the drum, allows the atomization and projection of liquid/slurry coating to the spinning seed mass. In villages, low-cost containers, such as earthen pots and buckets, can also be used for seed dressing by the farmers. This method can be used to apply a wide range of active materials, particularly chemical plant protectants. The rate of liquid chemical materials applied to seeds ranges from 0.05 to 1% of seed weight (Figure 3). Finishing powders or fluency powders are utilized shortly after the liquid coating to consume excess liquids, resulting in loading higher dosages of chemical seed dressing, especially modern pesticides [35].

#### 2.3.2. Film Coating

Film coating is known as a current method of encapsulating seeds with a thin layer of active materials, which comprises a mixture of polymers, pigments, plasticizers, and colorants [36]. The most popular seed coating apparatus is a rotary coater and fluidized bed but the former is used much more in commercial practice than the latter. Prior to application onto seeds, film coating polymers (liquid adhesive) are formulated to disperse or dissolve active components. This method produced nearly 90% application recovery [23], along with slight modification of the seed size, shape, and weight [24]. Generally, the increase of loading rates in seed weight is between 2 and 5% (Figure 3). To elevate loading from 5 to 8%, dry finishing powders can be added to the rotary coater or utilized with the dry powder applicator, a coating device where a rotating stainless steel brush sifts a powder material through a metering screen (Figure 2). Additionally, film coating promotes seed flowability during seed treating and sowing operations while eliminating the production of dust-off. Therefore, film coating has been obtained in use as a seed coating technique in the seed industry, in particular certain high-value vegetable species and other important crop seeds due to the excellent delivery of protectants on valuable seeds. When compared with other seed coating types, the significant advantages of film coating are the reduction of loss in the active ingredients throughout seed handling and transportation [35], as well as the prompter release of active components from the seed.

#### 2.3.3. Encrusting

Encrusting is another method of covering seeds that includes applying liquids and solid materials to create a completely coated seed with the shape of the initial seed maintained [37]. The two most ordinary encrusting machines to generate encrusted seeds are the rotary coater or rotating pan (Figure 2). As the earliest seed encrusting machine, the rotating pan is popularly composed of an inclined round pan driven by a rotating motorized pivot (Figure 2), where seeds are put in the pan on a slow spinning motion and then materials are gradually added to improve encrusted product size [38], followed by size sorting with sifters and then drying [24].

This procedure results in a smoother surface and a more uniform shape and promotes the weight and size of seeds, which can be utilized both in the field and in the greenhouse, thereby exploiting seed planting productivity [39]. Generally, the weight increase or build-up increase after the coating is more than film coating and ranges from 8–500% (Figure 3). Moreover, before packaging and storage, the freshly encrusted seeds need to be dried back to their original seed moisture content due to the addition of substantial amounts of water during the coating. This approach is commonly used on plants that gain benefit from seed singulation but do not require thinning during the post-emergence [40].

#### 2.3.4. Pelleting

Pelleting is the technique with liquids and inert materials (such as diatomaceous earth, talc, and bentonite) applied to the seed surface, enabling a greater weight increase, and is thus regarded as a continuation of encrusting operation [26]. Therefore, this process is also carried out using a rotating pan or rotary coater (Figure 2). Nonetheless, pelleting usually modifies seed morphology into a larger and spherical shape that is difficult to discriminate from the original seed shape [24]. Pelleting contributes to precise metering and improving plantability with mechanical planting equipment by increasing the percent weight up to 500–5000% (Figure 3). It facilitates making planting easy and accurate for small-seeded or irregular-shaped horticultural crops, including lettuce [41] and tomato [42].

Various forms, i.e., liquids and dry powders, serve as a binder. The mixture of dry powders and filler materials is to form a coating blend, with only water functioning as a solvent during the pelleting procedure [43]. It is crucial to select the appropriate liquids and fillers to make sure that pelleted seeds germinate without the constraints of the pellet matrix [37]. Similar to the encrusting process, the freshly pelleted seeds must be dried before packing and storing. In comparison with other coating methods, pelleting requires more time and expertise due to the extensive application of liquids, active ingredients, and solid powders. Additionally, the integrity of pelleting relies on the materials utilized (e.g., fillers and binders) and suitable techniques [23]. Unfortunately, manufacturers keep materials and methods of pelleting as closely guarded secrets, hampering the progress of pelleting technology [32].

#### 2.3.5. Extruded Pelleting

Extruded pelleting is the approach of seed coating with the addition of seeds and a relatively large amount of materials to produce pellets of various shapes and sizes, usually cylindrical [25]. This method aims to promote seed germination and seedling growth for small-seeded species through the dough mixture of seed and a host of materials [44]. Extruded pelleting requires specialized application machinery, that is, an extruder (Figure 2), which is similar to an industrial pasta machine that makes pasta in the food industry [25,45]. This apparatus consists of tubing, auger, dies, and cutter with a rotary wire. The process is that seed and materials (liquids and solids) are thoroughly mixed to produce a “friable” dough, which could efficiently flow through the auger. As the dough is compacted against the wall of the machine, seed dough material is extruded through a round die and then cut by a rotary wire cutter. All previously described coating methods avoid doubles or agglomerates (one coated propagule contains two or more seeds), while extruded pelleting incorporates multiple seeds into a single delivery unit. Furthermore, the build-up increase in seed weight is beyond 500% (Figure 3) due to the application of sufficient materials.

Depending on site environments and plants, supporting additives incorporated into the dough mixture contain water-sensitive binders, hydrophilic clay fillers, super absorbent polymers, plant growth regulators, and fertilizers [45]. As compared with standard seed coating techniques, extruded pelleting allows for several times more materials to be added to the seed surface. Likewise, precise placement with one or multiple seeds can be accomplished after the extrusion, which is conducive to the reduction of seed waste and control of the desired seed density per extruded pellet [33].

## 3. Seed Coating Agents

A broad range of materials in the process of seed coating are used as seed coating agents. According to materials function, these materials can be generally broken down into three primary categories: binders, fillers, and active ingredients [1]. Binders and fillers are used to provide for the thermal, physical, and mechanical characteristics of coated seeds, while active ingredients target the protection and increment of seed germination and seeding development [25]. It is worth noting that fillers and binders should be compatible with active components and not pose threats to the ability of seeds to germinate and grow.

### 3.1. Binders

Binders, functioning as adhesives, adhere to the seed and provide structural support and retention for active compounds. Seed dressing and film coating usually require a binder, which commonly refers to natural or synthetic polymers, for instance, methyl cellulose [46], carboxymethyl cellulose [47], polyvinyl alcohol [48], and gum arabic [49]. Apart from the polymer, certain organic binders in the fine powder form are mixed with the small particle fillers to produce a sticky coating powder when water is applied. They are usually delivered in aqueous solution form during or toward the end of the coating process to stick the exogenous materials to seeds and lower the amount of dusting off in the final product [1]. Moreover, the application of various layers with the binder during the different stages of coated seeds could offer a physical buffer to avoid direct contact between the active chemicals and the seed or other active layers. Additionally, the quantity of adhesive materials employed alters the mechanical properties of encrusted coatings, for instance, compressive strength, integrity, and disintegration time after water immersion [50].

### 3.2. Fillers

Fillers are popularly bulking inert materials and allow for physical modification, which is commonly used in the process of encrusting and pelleting. They are applied as either a single component or a combination of various components, and the most commonly used are bentonite [42], lime [51], diatomaceous earth [52], and talc [53]. These fillers are normally mineral materials that are mined from the earth with minimal alteration apart from crushing to gain a small particle size used in seed coating. In addition to physical structure support, chemically inactive fillers can separate the seed from the active substances (i.e., fungicides, insecticides, herbicides), accordingly protecting seeds against phytotoxic chemicals. The choice and optimum of the proper fillers rely on successful seed pelleting, which does not interfere with seed germination [41]. Furthermore, other features, such as cost, availability, safety, origin and environmental influences, should also be taken into account when selecting the most appropriate filler materials [54].

### 3.3. Active Ingredients

The objective of a wider range of active ingredients incorporated into seed coatings is to improve seed survival, increase resistance to abiotic and biotic stresses, aid in germination and growth, and promote ultimate yield [31]. The role of active substances for protection or enhancement is dependent on the mode of their action [37]. The most commonly reported active components in coating agents include protectants, nutrients, growth stimulants, microbial inoculants, soil adjuvants, and markers [36,55]. A summary of some of the most common active ingredients used in seed coating agents and their form, source, advantages, and disadvantages is presented in Table 1.

Seed coating protectants are the most broadly applied group of ingredients for controlling predation and infection by pathogens and pests at the time of sowing. Fungicides, pesticides, nematicides, bactericides, and herbicides are classified as protectants [31]. Nutrient amendments in coatings mainly focus on the delivery of polymer micronutrients, for example, copper, manganese, and zinc [36], which are used to compensate for soil deficiencies in these trace elements. The combination of growth stimulants properties and their application through seed coating has the pronounced potential for enhancing plant performance [63]. There are various categories of growth stimulants, such as plant-derived proteins [43,64], protein hydrolysates and amino acids [50,76], carbohydrate derivatives [59], and herbal extracts [77]. There is increased interest and demand based on nutrient and growth stimulant seed treatments [1]. Coating the seeds is considered a convenient and efficient tool for introducing beneficial microorganisms into the soil and consequently the rhizosphere of plant tissues [78]. There are three major types of plant growth-promoting microorganisms (PGPM) that are regarded as beneficial for plant nutrition: plant growth-promoting bacteria (PGPB), arbuscular mycorrhizal fungi (AMF), and rhizobia. Additionally, external environmental stresses may occur post-sowing due to drought, chilling, and heat, and the selection of seed treatments with natural and chemically synthesized may be exploited in the seed coating to mitigate these stresses.

However, the majority of existing chemical materials have a neutral or even detrimental effect on seed vigor, germination, and plant establishment [79] and are harmful to the environment [57], along with difficulties in biodegradation [80]. Therefore, it is necessary to explore innovative seed coating materials that are environmentally friendly. Biochar, as a novel material, may be expected to enhance seed germination and stand establishment, simultaneously ensuring agricultural sustainability, environment, and food safety. Due to the unique properties of biochar, i.e., high porosity and water holding capacity, the elevated contents of organic carbon and nutrients, it is proven to be a beneficial agent in seed coating formulations to increase germination and survivability [81].

### 3.4. Biochar

**Figure 4:**
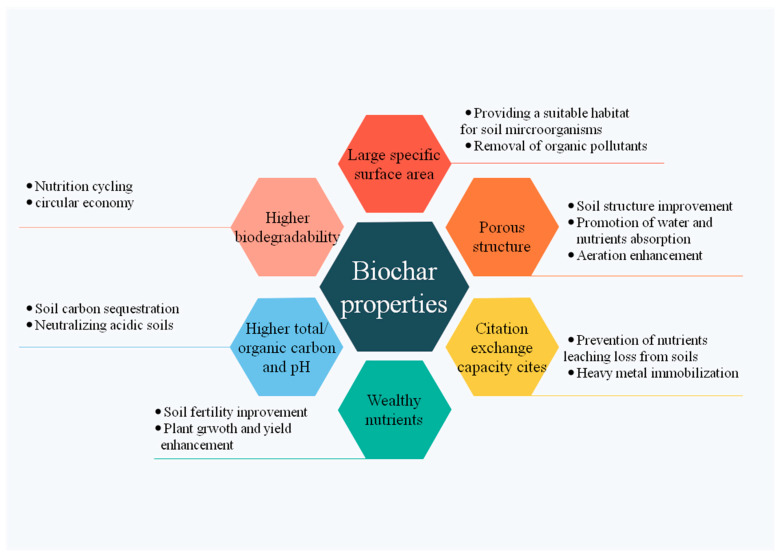
Properties of biochar and associated benefits.

Biochar is an environmentally persistent organic matter with rich carbon, which is prepared under anaerobic conditions by the pyrolysis of biomass [82]. Due to the large variability in the production feedstocks and pyrolysis conditions, biochar could possess broadly various physical and chemical properties (Figure 4). The most significant chemical difference between biochar and other organic matter is the much higher proportion of condensed aromatic structures and aromatic carbon, which is the key reason for the high stability of biochar. In terms of the components of biochar elements, the organic ingredients contain carbon majorly, whereas the inorganic portion could be composed of diverse micro- and macroelements, such as inorganic carbonates, potassium, sodium, magnesium, calcium, copper, zinc, iron, etc. Biochar is generally characterized by the large specific surface area, porous structure, high citation exchange capacity, and surface functional groups [83], which have direct links to the adsorption capacity of biochar.

Due to the above-mentioned properties of biochar, it can be used not only as a renewable fuel but also as an additive to improve soil quality. Furthermore, biochar displays great prospects for retaining soil fertility and promoting a circular economy in agriculture as a result of its higher biodegradable property. Recently, researchers are keen to utilize the biochar for its multifunctional role in agriculture (soil remediation [84], crop productivity [85]), ecosystem (carbon sequestration [86], wastewater management [87]), and energy (catalyst for biodiesel production [84]). The large-scale application of biochar is increasingly used for soil amelioration agents. Biochar amendment has substantial outcomes on the physical and physicochemical properties of soil, which directly induces soil nutrient dynamics and affects plant growth. The porous shape and high surface of biochar alter the soil’s physical nature by producing inter pores between soil and biochar particles, triggering a promotion in the total specific surface area of soil, accordingly increasing the porosity and aeration of the soil as well as water holding capacity. Biochar has numerous nutrients, organic matter, and ash content that can help in promoting soil organic carbon, cation exchange capacity, and soil pH for optimum plant growth [88]. In addition to affecting soil nature, biochar also stimulates the growth of rhizosphere microorganisms and mycorrhizal fungi [89]. Biochar particles have a high surface area and porous structure which promotes the colonization of microbes and improve soil microbial processes by increasing soil pH in acidic soils [90]. The addition of biochar induces changes in soil physical structure, altering gas transport and hydrodynamics, which facilitate the living behavior of soil biota [89]. These structural and functional transformations in soil properties under biochar addition can also significantly influence soil enzyme activities. Thus, biochar is a prospective material used for the improvement of soil quality and the simulation of soil microorganisms.

In recent years, based on the strong adsorption, water retention and organic nutrition characteristics of biochar, the usage of biochar with a dual role in active components and solid materials to coat crop seeds has obtained successful research progress and outcomes. A previous patent of biochar-based seed coating has revealed the coating method that mixing the seeds with starch or a suitable binder solution in a rotary tumbler and then adding the biochar material to the seed/binder mixture to achieve the desired thickness of seed coating [91]. Notably, this invention emphasized the necessity of biochar pretreatment in order to (i) improve the ideal properties like nutrient and moisture retention, (ii) remove harmful impurities from material pores, (iii) eliminate variations in properties due to derivation from various biomass materials, and (iv) load the appropriate active ingredients on the biochar particles. Another later study on biochar-based seed coating in combination with PGPM reported enhanced seedling growth when soluble phosphorus was applied, which could be interpreted that the water-holding characteristics of biochar may be a more pronounced factor in the observed plant growth promotion compared with the phosphorus solubilization effect from the inoculated bacteria [92]. Compared with regular pelleted seeds, biochar pelleting might improve the cost in the seed coating procedures due to the high production cost of biochar [93]. However, the incorporation of biochar usage in the seed coating helps efficient use of waste for a circular agriculture economy. Therefore, biochar-based seed coating is not only an exciting advancement in seed enhancement technology but also provides a novel approach to the recycling of organic waste.

## 4. Applications of Biochar-Based Seed Coating

Based on recent successful research in applying biochar seed coating techniques, the studies summarized in Table 2, report all recently available publications, including peer-reviewed literature and dissertations, which were searched on Google Scholar (https://scholar.google.com). Search terms included: “seed coat*”, “seed encrust*”, “seed pellet*”, “seed enhanc* techn*”, “seed enhanc* treat*”, “seed treat*”, “biochar”, and “activated carbon”. We included only papers that tested outcomes of seed coating with biochar or activated carbon and excluded reviews. The final dataset consisted of 25 articles where the publication dates ranged from 2014 to 2022 (Table 2).

Overall, seed coating with biochar has been successfully delivered to a diverse range of species of seeds, including both native and non-native species. The most investigated plant species concerning biochar coating were restoration species (twelve studies), such as Bluebunch wheatgrass and Wyoming big sagebrush, followed by legume species (eight studies), such as soybean and chickpea. The least studied species were vegetable (three studies) and cereal species (two studies). Likewise, the literature on biochar coating methods contained a higher number of studies focused on extruded pelleting (eight studies), followed by pelleting (six studies) and film coating (four studies). The outcomes of most studies we found, for instance germination, growth, protection, and yield, were commonly positive. Nine of the twenty-five instances focused on protection, with eight instances focused on germination, followed by four instances each between growth and yield. Recently, seed coating with biochar has received substantial attention for its ability to confer benefits to plant productivity and microbial inoculants. As the seed coating material, biochar is a desirous and cost-effective carbonaceous agent.

### 4.1. Seed Germination and Seedling Establishment Promotion

**Figure 5:**
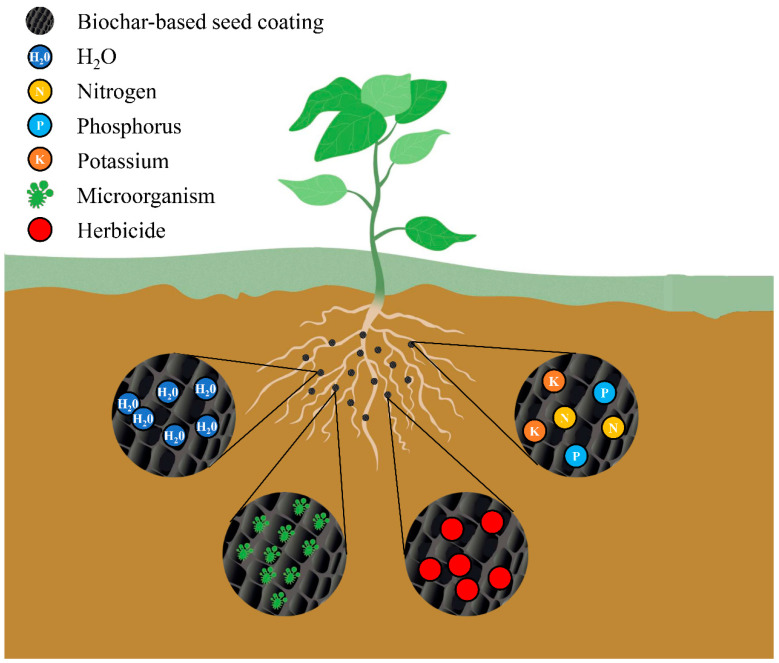
Mechanisms of biochar-based seed coating to promote plant growth.

Seed coating with biochar or activated carbon as the main active ingredient has been considered an important input in promoting seed germination and seedling establishment [45,102,103]. In a recent patent, Dong and coworkers [113] disclosed a method to develop safe and green seed coatings through novel seed coating formulation (e.g., biochar, sodium alginate, ethylene glycol, and bentonite) in lieu of traditional seed coating containing pesticides (such as carbofuran and carbendazim). The patent mentioned that biochar coating with 5–10% ratio displayed higher germination potential, root length, and root biomass accumulation than uncoated corn seeds. Madsen et al. [45] evaluated the effect of the incorporation of seed and biochar within an extruded pellet on Wyoming big sagebrush seedling emergence over a range of seedling depths. They found that pellets at all planting depths increased seedling emergence between 2.3 to 10.0 fold in the silt–loam soil. To screen proper binders for biochar-based seed coating, nine potential binders (such as methylcellulose, sodium hydroxide, starch, biobinder, etc.) were used to examine the effects of biochar-based coating on the plant germination and radicle extension of four species of crops and trees. The results indicated that biobinder and starch in the process of biochar coating performed optimally for the emergence and early seedling growth due to their ability to provide the structural support and retention of active materials, which can even expand the survival time of coating active components [103]. Also using the starch as a binder in the biochar-based coating formulations, Parvin et al. [94] found that seed coating with biochar (200 g/kg of seed) exhibited the highest germination rate, shoot length, and root length in comparison with untreated seeds of black gram. Similarly, Elamparithi et al. [102] reported that biochar coating in combination with organic materials recorded higher germination and seedling development compared with uncoated brinjal seeds. A recent study revealed that seed coating with a suitable ratio of markedly biochar-enhanced rice emergence and seedling stand [79]. This promotion in stand establishment can be due to the enhancement of water availability and rich nutrients around the seed, which is induced by biochar functioning, i.e., porous structure (Figure 5).

### 4.2. Plant Growth Enhancement

Biochar-based seed coating could be used to promote plant growth and production. Biochar addition can alter the soil conditions to affect plant growth positively, negatively, or neutrally. It has been proven that seed coating with biochar could be very effective at enhancing plant growth in many field crops [79]. For example, seed coating with biochar originating from corn stalk significantly increased plant height, leaf area, and leaf fresh weight in maize plants by 26.4%, 48.9%, and 19.8%, respectively, compared with non-seed coating [114]. In addition, Zhang [115] conducted pot and field trials to determine the impact of biochar coating on rapeseed growth and production. The author observed that coating treatment greatly improved crop height, leaf number, and dry biomass weight under both growth conditions, which eventually boosted rapeseed output. Recently, a study evaluated the efficacy of biochar derived from rice straw via seed coating on water-saving and drought-resistance rice [79]. Rice seeds coated with various proportions of biochar were sown in the growth chamber and open field to identify the appropriate ratio of biochar, and the effect of biochar coating on crop growth and yield under a dry direct-seeded system. Growth chamber trials conducted on rice seeds showed pronouncedly improved seedling growth when the content of biochar was 20% in the seed coating formulation. Moreover, biochar-coated rice was conducive to increasing the development of seedling roots [79]. Early and vigorous crop stands frequently capture more moisture and nutrient resources due to a greater root system and possess higher tiller numbers and larger leaf areas with enhanced photosynthetic products to maximize yields.

### 4.3. Suitable Carrier for Microbial Inoculants

Microbial seed coating is defined as a precise and efficient technology in which PGPM, e.g., PGPB, rhizobia, and AMF, are applied to the surface of plant seeds with the assistance of the binder and filler functioning as a carrier to promote plant growth and production and resistance to abiotic or biotic stress [31,36]. Biochar could be a suitable and promising alternative carrier for microbial inoculants. The high porosity of biochar may hold soil water and elevate aeration, which may offer a favorable habitat for bacterial proliferation [116,117]. Additionally, biochar rich in organic carbon and nutrients can serve as a carbon source that aids in the survival of microbial strains as a food source [101].

Generally, an inoculant formulation with seed coating is typically comprised of PGPM inoculants, suitable carriers, and multiple additives [36,118]. It has been extensively reported that biochar-based seed coating as a carrier could help maintain the viability and abundance of beneficial microorganisms [81,101,119]. For instance, Glodowska et al. [92] first proposed that biochar could function as a suitable carrier for *P. libanensis*, a phosphorus solubilizing bacteria, to generate inoculated seed coatings of corn. This suggested that biochar might be an alternative to peat moss, a non-renewable resource, and a standard carrier of inoculum. Thereafter, another study of theirs found the survival of *Bradyrhizobium japonicum*, a nitrogen-fixing bacteria, was extremely influenced by the physical and chemical characteristics of biochar [100]. Moreover, biochar-based coating with microbial inoculum allowed for the sustainment of a high bacterial population for long periods (9 months), thereby boosting the efficient nodulation of soybean and biological nitrogen fixation. Similarly, a pot experiment on the evaluation of the *Rizobia* inoculant carriers was conducted by Ghazi [97], who also observed a higher population of microbial inoculants 6 months after inoculation with biochar as a carrier substrate. Moreover, biochar produced from rice straw was found to ameliorate the antimicrobial effect of seed diffusate for kidney bean.

The effectiveness of biochar as a carrier on microorganisms survival varied with biochar feedstocks. Egamberdieva et al. [101] investigated the survival of *Bradyrhizobium* sp. in hydrochar from maize silage and biochar from wood, revealing that hydrochar sustained a higher population in comparison with biochar from wood. Meanwhile, another study reported that four types of biochar, two from hardwood feedstock and two from softwood, were tested for bacterial survival via seed coating. The findings indicated that pyrovac biochar from softwood and dynamotive biochar from hardwood was the most appropriate carrier due to the retention of a higher rate of bacterial survival [100]. Biochar prepared through the slow pyrolysis of agricultural wastes, such as leguminous plants and leafy vegetables, greatly enhanced the viability of two PGPM, *Bacillus* sp. strain A30, and *Burkholderia* sp. strain L2 [81]. Biochar-based formulations showed sustainment of the viable numbers of bacterial cells up to 180 days of storage, while seed germination trials observed a pronounced increase in the germination of coated seeds in comparison with uncoated controls. Notably, the properties of biochar, for instance, pyrolysis temperature, pH, degree of oxidation, and granulate size also influence microbial survival and colonization [119].

#### 4.3.1. Biochar-Based Microbial Inoculants and Crop Growth

Previous studies indicated that microbial seed coating with biochar as a carrier could promote crop growth and output [36]. Tripti et al. [81] investigated the potential of bioformulations containing mixtures of biochar originated from agriculture waste and fly ash (a byproduct of the coal burning process) containing PGPM to coat tomato seeds. Biochar-based formulations with *Burkholderia* sp. strain L2 improved shoot growth (an increase of 61%, 73%, and 46% in maximum length, fresh biomass, and dry biomass, respectively), root growth (an increase of 114%, 141%, and 99% in maximum length, fresh biomass, and dry biomass, respectively), and yield (an increase of 225% in fruit yield), respectively, compared to uncoated controls. Similar observations were recorded by Camara-Williams [99], where seed pelleting with groundnut husk biochar increased mean nodule numbers and nodule fresh and dry weight of soybean under the pot experiments and field experiments.

Biochar-based seed coating with PGPM can be particularly pertinent in low-input and organic agricultural systems due to its potential to reduce the inputs of agrochemicals and improve crop productivity. Rocha et al. [95] showed that a biochar-based seed coating was a successful tool to inoculate the AMF isolates and rhizobacteria that results in significant increases in shoot biomass (76%), the number of pods and seeds per plant (52% and 56%, respectively), and grain yield (56%) in cowpea under no fertilization field conditions. By using a similar coating material and formulation, Rocha et al. [96] investigated the influence of biochar-based seed coating on chickpea production under greenhouse and field conditions. They observed that biochar coating with multiple AMF isolates displayed higher shoot biomass (14%) and seed individual weight (21%) under greenhouse conditions and increased pod (160%), seed numbers (148%), and grain yield (140%) in the natural fields without receiving fertilizers.

#### 4.3.2. Biochar-Based Microbial Inoculants and Crop Nutrition

In addition to the enhancement of plant growth, biochar-based seed coating with microbes is a promising and practical approach for improving the nutritional value of various crops [119]. For example, Egamberdieva et al. [101] evaluated the response of lupin to the biochar-based inocula of *Bradyrhizobium* sp. in pot experiments under irrigated and drought conditions. They found that biochar-based coating produced plant nutrient concentration increments of 39%, 31%, 34%, and 21% for nitrogen (N), phosphorus (P), potassium (K), and magnesium (Mg), respectively, compared to the control under water deficit. Similar results were reported by Glodowska et al. [92], where biochar coating with *P. libanensis* promoted chlorophyll content and N uptake of corn shoots when soluble P was applied. In the subsequent study, Glodowska et al. [100] observed a significant increase in N (90%), carbon (C, 8%), and N/C (100%) of soybean plants in the biochar seed coating with *Bradyrhizobium japonicum* under N-free nutrient solution. Such improvements could be attributed that biochar protects functional microbes living against environmental stresses by providing a favorable microenvironment [116,117]. The underlying mechanisms by which biochar-based seed coating enhances plant growth and nutrient uptake mainly include moisture absorption and retention, nutrient provision and mobilization, beneficial microorganisms recruitment, and herbicide adsorption to protect the native species (Figure 5).

### 4.4. Herbicide Selectivity Increase

There are limited cost-effective strategies for successfully restoring native species in areas where exotic species are dominant. This is because non-native species commonly outcompete native species, which is reflected in the higher plant, seed bank densities, and growth rates of non-native species [12]. These invasive species are therefore removed or controlled as part of ecological restoration to avoid further unfavorable outcomes, for instance reducing the survival of native species [120], modifying vegetation structure [121], and altering soil nutrients [122]. It has been widely proposed that seed coating containing biochar or activated carbon to provide herbicide protection could facilitate controlling invasive species and achieve successful restoration, since coating treatments are applied simultaneously with herbicide application [104,108,112]. The herbicide protection pods (HPPs) technology was developed by Madsen et al. [108], designed to combine the protective ability of activated carbon banding with the selectivity of seed coating. This method is anticipated to provide sufficient coverage of activated carbon for the seeded species to neutralize herbicide adsorption while minimizing herbicide protection to weed species. For example, *Elymus elymoides* (bottlebrush squirreltail) and *Agropyron fragile* (Siberian wheatgrass) seeds incorporated into biochar extruded pellets displayed greater density than the control seedlings (bare seed) when sites were treated with imazapic (a herbicide), which effectively controlled exotic annual weeds [112]. Similarly, bluebunch wheatgrass seeds based on biochar coating successfully grew in a laboratory environment treated with pre-emergent herbicide, where the herbicide effectively controlled invasive annual grasses [106]. Recently, Terry et al. [105] evaluated two approaches, namely biochar coating and furrow, simultaneously with an imazapic application to control annual grasses. They found that combining biochar coating and furrow treatment mitigated harmful herbicide effects on seedling emergence, plant densities, and growth. The results showed that coating seeds with biochar could assist native species to achieve a similar establishment as a non-herbicide seeding with a lower abundance of exotic grasses when in combination with agronomical practices. Remarkably, more studies are required to investigate these techniques on other plant species, soil types, and climatic conditions before they are large-scale applied in the restoration. After all, some species are more susceptible to pre-emergent herbicide [123], causing the loss of seedling vigor due to inadequate protection from seed coatings and deep furrows.

## 5. Future Prospects

Extensive reports showed that biochar-based seed coating contributed to the improvement of stand establishment and plant production due to the distinct physiological and biochemical properties of biochar. Nevertheless, not all studies show that biochar seed coating could stimulate seed germination. Xavier et al. [98] and James [18] observed that seed coating with biochar had no effect on the seed and seedling performance compared to uncoated seeds. Despite some uncertainty, it is worthy to further evaluate the effectiveness of biochar seed coating on different plant species under various environments concerning biochar types [54]. For instance, some local initiatives (e.g., “biochar seedballs” in Kenya) are assessing the impacts of this approach on trees and grass species, aiming to rehabilitate degraded land damaged by human activity [124]. Below we present key and unresolved points that future researchers on biochar-based seed coating should strive to focus on:

(1) Further research is required to affirm the utility of biochar-based coating with a focus on biochar pretreatment to eliminate harmful substances. Most of the existing literature maintains a commonly favorable outlook on the application of biochar via seed coating on multiple aspects. However, a study was performed by Williams et al. [125], who found that biochar-based seed coating had either a neutral or negative effect on seed germination and plant establishment for all plant species. The authors hypothesize that some volatile organic compounds in biochar may negatively affect seed germination. Likewise, previous studies have raised concerns about the toxic substances (carcinogenic polycyclic hydrocarbons, heavy metals, and harmful free radicals) present in biochar. Zhang et al. [126] explored the impact of biochar nanoparticles on seed germination and seedling growth of three plants, including rice, tomato, and reed, indicating that the risk of biochar nanoparticles toxicity was determined by the biochar feedstock. Despite this, biochar nanoparticles are the promising agents of seed priming for future research.

(2) Future research on biochar-based seed coating could be developed on the novel and efficient seed coating formulation by the varying biochar feedstocks, coating ingredients, and coating techniques. The efficiency of biochar-based seed coating depends on the types, properties, and preparation methods of biochar. For example, most biochar were commonly alkaline, and high alkalinity (pH > 9) may have adverse influences on crop emergence, particularly at higher biochar ratios (>30%) [79]. Thus, more studies comparing various biochar origins with different crops should be further explored. Moreover, more clarity regarding the ingredients and methodological details of seed coating with biochar is required.

(3) So far, biochar-based seed coatings have been mainly designed for the ecosystem restoration of native plant species, such as Bluebunch wheatgrass and Wyoming big sagebrush. More attention should be paid to other agricultural species (i.e., fruit/vegetable, oil crops, and fiber crops) in biochar-based seed coating, particularly for sustainable farming. The optimum and development of biochar-based seed coating for a wide range of crops could transform agricultural production systems to be more sustainable. Aerial seeding may be an effective way to restore ecosystems owing to rapid seed delivery over large areas [54]. Yet, biochar-based coating combined with aerial seeding (i.e., unmanned aerial vehicles) has rarely been used for ecological restoration. Additionally, the scarcity of studies reported on the roles of biochar-based seed coating in ameliorating abiotic stresses (such as salinity, chill, and heat) of plants. So, the effects of biochar-based seed coating on seed germination, plant growth, and stress resistance under various growth conditions should be conducted. Future research on the economic assessment for the production of biochar-based seed coating is also included.

(4). On the other hand, with the application of biochar via seed coating, the interaction mechanisms of plant–microbe–soil become of great importance and are promising lines of study for promoting the efficiency of biochar-based seed coating in the field. Although biochar may offer a favorable habitat for bacterial proliferation, the effects of biochar-based seed coating on the microbial abundance in the rhizosphere are still unclear. Therefore, it is necessary to understand the behavior of rhizosphere microbes recruited by biochar in the seed coating under different environmental conditions by the soil microbiome. The metagenomic of soil microorganisms approach will provide new avenues to address the issues of biochar-based seed coating, along with bringing novelty in revealing the interacting mechanisms of plant–microbe–soil.

## 6. Conclusions

Erratic germination and plant establishment led to the loss of crop production due to adverse environmental and biological factors. Seed coating has the potential to be an efficient and cost-effective approach for alleviation of stresses, promotion of crop yield, and provision of desirable merits to the seeds via the application of external materials. Yet, the current materials of seed coating are still facing limitations. Some coating agents were hard to biodegradation, which causes damage to the environment. On the other hand, the chemical components in the seed coating had toxicity to seeds, reducing the seed vigor or hampering seed germination. Recently, biochar-based seed coating has gained much interest due to biochar-possessing high porosity and water holding capacity, as well as wealthy nutrients, and has been proven to be a beneficial agent in seed coating formulations. Previous studies showed that biochar-based seed coating was not only able to improve the emergence and growth of the seedling but also increase plant production and nutrition. Additionally, biochar via seed coating was extensively applied to the delivery of PGPM and the ecological restoration of native species.

## Figures and Tables

**Table 1:** Review of active ingredients in the seed coating agents.

Types of Active Ingredients	Active Ingredients	Ingredient Form	Ingredient Source	Ingredient Benefits	Ingredient Flaws	References
Protectants	Fungicides, insecticides, nematicides, bactericides, predator deterrents, herbicides	Variable	Synthetic chemicals	Protect seed; promote germination and growth	Ecosystem contamination	[56,57,58,59]
Nutrients	Macronutrients: P, K; micronutrients: boron, copper, manganese, zinc	Solid	Synthetic chemicals; mineral	Improve growth and yield; alleviate trace elements deficiency in soil	Reduction in germination and emergence caused by nutrients	[22,60,61,62]
Growth stimulants	Phytohormone: salicylic acid, gibberellic acid, auxin; protein hydrolysates: soy flour	Solid	Synthetic chemicals; natural products or derivatives	Stimulate germination, improve growth, development, and stress resistance	Sometimes delay germination and hamper root growth	[63,64,65,66]
Microbial inoculants	Arbuscular mycorrhizal fungi, plant growth-promoting bacteria and rhizobia	Solid	Natural products	Promote growth, yield and nutrition; increase abiotic and biotic tolerance	Poor survival of the inoculant; the insufficient amount of microbial inoculant for small seeds	[22,31,35,36,67]
Soil adjuvants	Soil hydrophilic materials or hydro absorbers: hydrogels; soil surfactant	Solid	Synthetic chemicals	Accelerate or delay germination through influencing water absorption	Environment risk due to the difficulty of degradation; sometimes unaffected germination or seedling growth	[1,65,68,69,70,71]
Markers	Fluorescent dyes and magnetic powder	Solid	Synthetic chemicals	Improve the traceability of seed batches; limit the predation of seed by birds	Inconvenient application; requires sophisticated detection equipment	[72,73,74,75]

**Table 2:** Studies on the application of seed coating with biochar published in peer-reviewed studies and dissertations.

Species	Biochar Sources	Coating Materials	Active Ingredients	Coating Equipment	Coating Type	Main Findings	Reference
Rice	Rice straw	Biochar, talc, attapulgite	Biochar	Rotary coater	Pelleting	Biochar seed coating improved the emergence rate and seedling growth both under laboratory study and field study.	[79]
Corn	Hardwood and softwood	Biochar, peat moss, P. Libanensis, guar gum	P. Libanensis	Nd	Film coating	Biochar-based seed coating with inoculants enhanced germination speed and corn growth without increasing the phosphorous content of the plants.	[92]
Black gram	Green waste of Prosopsis plant	Biochar, starch	Biochar	Nd	Nd	Biochar coating @ 200 g/kg of seed performed well and increased the seedling growth, vigor and biochemical parameters.	[94]
Cowpea	Nd	Pseudomonas libanensis TR1, Rhizophagus irregularis, biochar, gum arabic	Pseudomonas libanensis TR1, Rhizophagus irregularis	Rotating pan	Seed dressing	Seed coating with PGPR and multiple AM fungal isolates increased shoot dry weight (76%), grain yield (56%) and grain lipid (25%).	[95]
Chickpea	Nd	R. Irregularis inoculum, biochar, gum arabic	Multiple Rhizophagus irregularis isolates	Rotating pan	Seed dressing	Plants inoculated with multiple isolates in the greenhouse displayed higher shoot dry weight (14%) and seed individual weight (21%), while in the field increased pod (160%), seed number (148%), and grain yield (140%).	[96]
Kidney bean	Rice straw	Peat moss, vermiculite, biochar	Rhizobium phaseoli	Nd	Nd	The biochar-based carrier inoculant gave the highest nodule dry weight, plant dry weight, plant height.	[97]
Stylosanthes cv. Campo Grande	AC	Dolomitic limestone, sand, polyvinyl acetate, AC, calcium silicate	Calcium silicate	Rotating pan	Pelleting	Coating with calcium silicate and polyvinyl acetate was outstanding for germination speed index and fresh and dry matter of shoot and root.	[98]
Soybean	Saw dust, groundnut husk, rice husk and rice straw	Biochar, rock phosphate, calcium carbonate, rhizobium inoculants	Biochar	Nd	Pelleting	Seed pelleting with biochar improved mean nodule number, nodule fresh weight and nodule dry weight in pot and field experiments.	[99]
Soybean	Hardwood and softwood	Biochar, peat moss, Bradyrhizobium japonicum, guar gum	Bradyrhizobium japonicum	Nd	Film coating	Biochar solid inoculant positively affected plant growth metrics, root characteristics, and the chemical composition of plants supplied with N-free nutrient solution.	[100]
Lupin	Maize silage, maize and wood	Hydrochar, pyrolysis biochar, Bradyrhizobium sp. (BR) inoculants	Bradyrhizobium sp.	Nd	Film coating	HTC-based BR Inoculants significantly enhanced plant growth, N and P uptake, and lupin nodulation under drought conditions than BR strain inoculation.	[101]
Brinjal	Nd	Sargassum sp., Kappaphycus sp., azophos, biochar, talc powder	Biochar	Nd	Pelleting	Seed pelleting recorded higher seed quality and biochemical parameters.	[102]
Radish, lettuce, coreopsis, white birch	Nd	Pectin, DTL 35% (lignin), methylcellulose, sodium hydroxide, starch, biobinder, biocoat	Biochar	Nd	Film coating	Seed coating with bio-based compounds and starch binder performed best for seed germination, radicle extension, and non-phytotoxicity.	[103]
Tomato	Agricultural wastes	Flyash, biochar, Burkholderia sp., Bacillus megaterium	Burkholderia sp., Bacillus megaterium	Nd	Nd	The biochar based bioformulation using Burkholderia sp. Tremendously enhanced the productivity of tomatoes and soil fertility. A mixture of biochar and flyash inoculated with Burkholderia sp. Showed noteworthy results for the growth parameters and yield of tomato.	[81]
Crested wheatgrass	AC	AC, Ca Bentonite, worm castings, compost, super absorbent powder, super absorbent fine granules	AC	Extruder	Extruded pelleting	HPPs increased the abundance of crested wheatgrass (300%) compared with bare seeds.	[104]
Bluebunch wheatgrass	AC	AC, polyvinyl alcohol	AC	Rotary coater	Extruded pelleting	Combining carbon coatings and furrow treatments mitigated harmful herbicide effects on seedling emergence, plant densities, and growth.	[105]
Bluebunch wheatgrass	AC	AC, compost, worm castings, bentonite clay, polyvinyl alcohol	AC	Rotary coater	Pelleting	The AC-based coating produced 307% higher emergence, 235% higher seedling height, and 87% higher biomass than uncoated seeds with herbicide application, respectively.	[106]
Bluebunch wheatgrass	AC	Bentonite clay, polyvinyl alcohol, host pepper, cayenne pepper, anthraquinone, methyl-nonyl-ketone, pine needle essential oil, bergamot essential oil, neem oil, AC, beta cyclodextrin	AC	Rotary coater	Encrusting	Seeds coating with ghost pepper, neem oil, and AC reduced rodent seed-predation by 47–50% than control.	[107]
Bluebunch wheatgrass, downy brome	AC	AC, diatomaceous earth, polyvinyl alcohol	AC	Extruder	Extruded pelleting	Coating with AC was 4.8-, 3.8-, and 19.0-fold higher than untreated seeds in density, height, and biomass, respectively, at the higher levels of herbicide application.	[108]
Wyoming big sagebrush	Western juniper tree	Calcium bentonite, biochar, worm castings, compost, super-absorbent polymer, starch, surfactant, cytokinin, gibberellic acid, indole butyric acid	Nd	Extruder	Extruded pelleting	Extruded pellets improved seedling emergence between 2.3- to 10.0-fold in the silt–loam soil.	[45]
Wyoming big sagebrush, bluebunch wheatgrass	AC	Ca bentonite, AC, worm castings, compost	AC	Extruder	Extruded pelleting	HPPs protected seeded species at low, mid, and high rates of the preemergent herbicide, and had a greater abundance and size of plants compared to bare seeds.	[109]
Wyoming big sagebrush, sandberg bluegrass	AC	Bentonite clay, AC, compost, worm casting fines, fungicide	AC	Extruder	Extruded pelleting	The smaller HPPs produced about two-fold higher final emergence and higher aboveground biomass than larger pellets and maintained protection from herbicide toxicity.	[110]
Lolium rigidum, annual ryegrass	AC	AC, bentonite, diatomaceous earth, sand, starch, water-holding crystals	AC	Extruder	Extruded pelleting	Extruded pellet formulation increased seedling tolerance to pre-emergent herbicide with mortality reduced from 96% in non-pelleted seeds to 22% in pellets containing AC.	[111]
Bottlebrush squirreltail, Siberian wheatgrass	AC	Ca Bentonite, AC, worm castings, compost, superabsorbent powder, super absorbent fine granules	AC	Extruder	Extruded pelleting	These two bunchgrasses had greater density and growth (height, leaf length, number of stems, and leaves) when incorporated into AC pellets compared with bare seeds.	[112]
California brome, blue Wildrye	Beetle-killed pine trees	Biochar, CaCO_3_, hydrophilic polymers, micronutrients, GA_3_	Biochar	Nd	Nd	Seed coating showed equal or slightly higher germination over the untreated control.	[18]
Mountain brome, prairie junegrass, Wyeth’s buckwheat, western yarrow	Beetle-killed ponderosa, lodgepole pine	Biochar, polyvinyl alcohol	Biochar	Rotating pan	Pelleting	Biochar seed coatings had either a neutral or negative effect on germination and growth under different temperature and moisture conditions, and slightly improved mountain brome root weight and prairie junegrass cover in the field.	[107]

Notes: Nd, not described; AC, activated carbon; HTC, hydrochar from maize silage; HPPs, herbicide protection pods containing AC.
